# Relative quantification of phosphoproteomic changes in grapevine (*Vitis vinifera* L.) leaves in response to abscisic acid

**DOI:** 10.1038/hortres.2016.29

**Published:** 2016-06-22

**Authors:** Supakan Rattanakan, Iniga George, Paul A Haynes, Grant R Cramer

**Affiliations:** 1 Department of Biochemistry and Molecular Biology, University of Nevada, Reno, Reno, NV, USA; 2 Department of Chemistry and Biomolecular Sciences, Macquarie University, North Ryde, New South Wales, Australia

## Abstract

In a previous transcriptomic analysis, abscisic acid (ABA) was found to affect the abundance of a number of transcripts in leaves of Cabernet Sauvignon grapevines with roots that had been exposed to 10 μm ABA for 2 h. Other work has indicated that ABA affects protein abundance and protein phosphorylation as well. In this study we investigated changes in protein abundance and phosphorylation of Cabernet Sauvignon grapevine leaves. Protein abundance was assessed by both label-free and isobaric-label quantitive proteomic methods. Each identified common proteins, but also additional proteins not found with the other method. Overall, several thousand proteins were identified and several hundred were quantified. In addition, hundreds of phosphoproteins were identified. Tens of proteins were found to be affected in the leaf after the roots had been exposed to ABA for 2 h, more than half of them were phosphorylated proteins. Many phosphosites were confirmed and several new ones were identified. ABA increased the abundance of some proteins, but the majority of the proteins had their protein abundance decreased. Many of these proteins were involved in growth and plant organ development, including proteins involved in protein synthesis, photosynthesis, sugar and amino-acid metabolism. This study provides new insights into how ABA regulates plant responses and acclimation to water deficits.

## Introduction

Grapevines (*Vitis vinifera* L.) are an economically important fruit crop worldwide. They are used for the production of wine, table grapes, juice and raisins, and are worth millions of dollars every year for the US industry. Abiotic stresses affect both quality and quantity of grape production.^1,2^ Mild drought stress or the application of abscisic acid (ABA) increase phenolic compounds such as anthocyanin, catechin and quercetin in the fruit^3–5^ and, in part because of their antioxidant activities, can benefit human health. Severe water deficit can reduce photosynthesis, inhibit vine growth, and decrease the quality of grapevines.^6^ Thus, there is an optimal level of drought stress that produces an optimal grape wine quality. A better understanding of grapevines responses to drought stress will allow one to minimize the loss of grapevine production and maximize grape quality.

ABA is a plant hormone that has important roles in developmental processes and adaptive stress responses in plants such as salt, cold and drought stress.^7,8^ ABA regulates plant responses by altering protein activities directly by post-translational modifications such as phosphorylation and nitrosylation, and indirectly by affecting the transcription of many genes.^9–11^ A model of ABA signaling has been constructed and involves a central core pathway of PYR/PYL/RCAR receptors, 2C-type protein phosphatases (PP2C) and SNF1-related protein kinase 2 (SnRK2).^12,13^ Several transcription factors (AREB/ABFs) and ion channel proteins (SLAC1 and KAT1) are phosphorylated by SnRK2 kinases,^14,15^ but very likely there are many more proteins to be identified. Moreover, there may be other kinases in the ABA signaling pathway that have yet to be discovered.^10,11^

Omic technologies have been used to gain better understanding of plant responses to stresses.^16^ Despite the great advances transcriptomic analyses have contributed to our understanding, there are far fewer proteomic and phosphoproteomic studies, which address a different level of plant regulation. Furthermore, recent studies from our lab indicate that the abundance of most proteins is not well correlated with transcript abundance.^17,18^

In a previous study, the transcriptomic responses of grapevine to ABA were examined.^19^ Some of the results from that study indicated that the roots, which had been treated with 1 μM ABA for 2 h had 538 significantly differentially expressed genes (DEGs), whereas the leaf from the same plant had 69 significantly DEGs in response to the root treatment. Genes with significantly increased transcript abundance in leaves were involved in protein folding and the protein amino-acid phosphorylation process in roots. In this paper, we extend this study by examining the proteomic and phosphoproteomic responses of the grapevine leaves of vines whose roots were treated with ABA.

In this study, we identify proteins and phosphoproteins involved in the ABA signaling pathway in grapevine. A label-free approach was first used to identify and quantify changes in protein abundance. In addition, we utilized a second approach, using 6-plex isobaric mass tagging technology, labeling peptides with structurally identical tags but different reporter ions. Our data sets revealed motifs and phosphorylation sites that are consistent with other plant phosphoproteomes.^11,20–24^

## Materials and methods

### Sample collection and ABA treatment

Rooted cuttings of Cabernet Sauvignon grapevines were grown in a growth chamber for 2 to 3 weeks before carefully transferring them to an aeroponic system located in a greenhouse under standard conditions (with supplemental sodium vapor lamp lighting (16 h light (minimum 400 μE m^−2^ s^−1^) at 28 °C and 8 h dark at 18 °C cycle). Each container (43.2 cm(*L*)×27.9 cm(*W*)×20.3 cm(*H*)) had a nebulizer with a fogger head size of 3.8 cm diameter×4.4 cm height for each experimental replicate (three containers for control and three containers for ABA treatment). The lid of each container had small holes large enough for several rooted plants to be passed through and into the container. Gibeaut’s solution^25^ was used to provide the macronutrients and micronutrients to the vines. The pH of the solution was maintained at 6.0. Root and leaf samples were grown for 3 months before treatment.

ABA was applied to the roots by continually misting the roots with 10 μm ABA added to the Gibeaut’s solution in the aeroponic system; leaves from the same vine (but not directly exposed to the misting solution) and exposed roots were collected after 2 h of root exposure with fresh control and ABA solutions. Root samples were quickly rinsed with tap water and collected leaf and root materials were rapidly frozen in liquid nitrogen before storage at −80 °C.

### Label-free approach

#### Proteome sample preparation and LC–MS/MS analysis

A phenol extraction protocol was used for *Vitis vinifera* leaves and is based on previous protocols (Vincent, Wheatley *et al.* 2006).^17,18^ Trypsin in-solution digestion, peptide extraction and fraction analysis by nanoflow liquid chromatography tandem mass spectrometry (LC–MS/MS) were conducted essentially as previously described.^26^ Briefly, three experimental replicates of ABA-treated leaves and untreated leaves were run separately on an LTQ Velos Pro mass spectrometer (Thermo, San Jose, CA, USA) for the sample-optimized gas phase fractionation. Chromatography was performed on an Easy-nLC II (Thermo) with magic C18 AQ column (3 μm bead size, 200 Å pore size, 0.1 mm inside diameter ×100 mm; Michrom Biosciences, Auburn, CA, USA). Each sample was analyzed in four 120-min LC–MS/MS runs at 0.5 μL min^−1^, each using a different precursor mass range (400–506, 501–658, 653–913 and 908–1600). The *m*/*z* ranges for four gas phase fractions per sample were optimized empirically by analyzing a mixture of pooled samples from *m*/*z* 400–1600, then creating gas phase fractionation fractions to approximately evenly distribute peptide observations among the four fractions.

#### Protein identification and quantification

A protein database for *V. vinifera* was combined as in a previous report (Cramer *et al.* ^18^). The X! Tandem and the GPM Cyclone (www.thegpm.org) in the automated mode using MudPIT merging were used for peptide-to-spectrum matching. Protein and peptide false discovery rates (FDR) were calculated using reverse database searching.^27^

Low-stringency protein identification data from individual replicate experiments were transformed into high-stringency data sets containing only reproducibly identified proteins using a suite of R modules known as the Spectral Counting Reporting Analysis Program (Scrappy).^28^ For a protein to be considered as reproducibly identified it needed to satisfy two criteria: it must be present with at least five peptides across all three replicates, an average of 1.7 peptides per experiment. The same program was used for calculation of protein abundance using normalized spectral abundance factors (NSAF), and determination of significantly differentially expressed proteins (*P*<0.05) based on Student's *t*-test across three replicate NSAF values.

### TMT labeling approach

#### Proteome sample preparation

The phenol protein extraction was used as mentioned above. The protein concentration was determined by using EZQ protein quantitation according to the manufacturer’s instructions (Bio-Rad, Hercules, CA, USA). Protein samples (10 mg) were digested with trypsin and desalted by Sep Pak Plus C18 cartridges (Waters Inc, Milford, MA, USA). Peptides (10 mg) were resuspended in 100 μL of 1% acetic acid.

#### Phosphopeptides enrichment and TMT labeling

The immobilized metal affinity chromatography (IMAC column; Qiagen Ni-NTA spin column (Cat No. 31014)) was loaded with 50 μL of peptides. The IMAC column was washed four times; twice with 1 bead volume of 0.1 m NaCl/25%ACN/0.1% acetic acid, once with 1 bead volume of 1% acetic acid, and once with half bead volume of ddH_2_O. Peptides were eluted with 3× bead volume using 6% NH_4_OH (total 120 μL); the elution was collected in a silanized glass insert, and then dried with a speed-vac. The tandem mass tags (TMT) labeling method was followed according to the manufacturer’s instructions (TMT Fisher # 90061, Thermo Fisher Scientific, Asheville, NC, USA). The untreated leaf samples were labeled with TMT-126, TMT-127, and TMT-128, while ABA-treated leaf samples were labeled with TMT-129, TMT-130, and TMT-131. All TMT labeling samples were combined before sample fractionation using hydrophilic interaction liquid chromatography according to Pucci *et al.* (Pucci, Giuliano *et al.* 2009). A total of 40 fractions were evaporated to dryness in a vacuum centrifuge and resuspended in 100 μL 5% CAN and 0.1% formic acid for LC–MS/MS analysis.

#### LC–MS/MS analysis

Digested peptide samples were analyzed using LC–MS/MS at the Nevada Proteomics Center (University of Nevada, Reno, NV, USA). The peptides were separated and analyzed using a Michrom Paradigm Multi-Dimensional Liquid Chromatography instrument (Michrom Bioresources Inc., Auburn, CA, USA) coupled with a Thermo LTQ Orbitrap XL mass spectrometer (Thermo Fisher Scientific, San Jose, CA, USA). Peptide samples were dissolved in 100 μL of 0.1% formic acid and loaded onto a ZORBAX 300SB-C_18_ 5-μm (5×0.3 mm) trap column (Agilent Technologies, Santa Clara, CA, USA), eluted from the trap, and then separated with a reverse phase Michrom Magic C_18_AQ column (3 μm, 200 Å, 0.2×150 mm) by a gradient elution using solvent A (0.1% formic acid) and solvent B (0.1% formic acid in ACN) at a flow rate of 2 μL min^−1^. The gradient was set from 5 to 40% solvent B for 90 min, increased to 80% solvent B in 10 s and held at 80% solvent B for 1 min. MS spectra were recorded over the mass range of *m*/*z* 400–1600 with resolution of 60 000. The three most intense ions were isolated for fragmentation in the linear ion trap using CID with minimal signal of 500 and collision energy of 35.0 or using HCD with a minimal signal of 1000, collision energy of 55.0, and an activation time of 30 ms. Dynamic exclusion was implemented with two repeat counts, repeat duration of 15 s and exclusion duration of 90 s.

#### Protein identification and quantification

All MS/MS samples were analyzed using Sequest (Thermo Fisher Scientific; version 1.0). Sequest was set up to search the uniprot_Vitis_20121010 database (54 242 entries). Search parameters included fragment ion mass tolerance of 1.00 Da and a parent ion tolerance of 10.0 p.p.m., trypsin enzyme specificity, carbamidomethylation of cysteine as a fixed modification and oxidation of methionine, phosphorylation of serine, threonine and tyrosine and TMT6plex of lysine and the N-terminus as variable modifications. Scaffold (version Scaffold_4.4.1, Proteome Software Inc., Portland, OR, USA) was used to validate MS/MS based peptide and protein identifications. Peptide identifications were accepted if they could be established at greater than 95.0% probability by the Peptide Prophet algorithm^29^ with Scaffold delta-mass correction. Protein identifications were accepted if they could be established at >95.0% probability and contained at least 1 identified peptide.

Relative abundances of proteins were calculated based on Dayon *et al*.^30^ Briefly, a normalization of the reporter intensities by the sum of all the reporter intensities was made in order to determine the relative abundance of each reporter. Then the log_2_ ratio of average TMT^129,130,131^/TMT^126,127,128^ (ABA/Control) were obtained from the average values. Finally, an unpaired *t*-test was assessed to test the significance of the abundance difference (*P*<0.0.5).

### Motif analysis

In order to identify potential enzyme recognition sites, Scaffold PTM scans the data set for over-represented patterns in the amino acids surrounding modification sites. It uses the iterative statistical method described in a previous publication.^31^ Sequence logos were generated using Weblogo^32^ of all phosphorylation sites with Ascores⩾13 (*P*<0.05). The frequency of each residue present in each data set is proportional to its height. The phosphorylation site is located at 0 on the *x* axis flanking by 12 amino-acid residues (at position −6 to +6).

## Results

### Proteomic changes in leaves of grapevine in response to ABA

**Figure 1:**
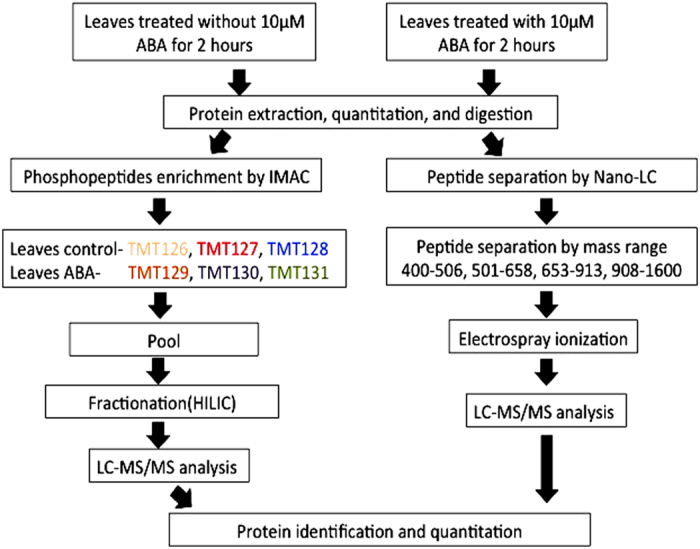
A workflow of a comprehensive large-scale MS-based proteomics and phosphoproteomics strategy. MS, mass spectrometry.

Leaves of Cabernet Sauvignon grapevines were collected after 2 h treatment with 10 μM ABA to the roots of the same vines. In this study, two approaches were employed to identify and quantify proteomic changes (Figure 1). The first method was label-free quantitative shotgun proteomics using nanoflow liquid chromatography-tandem mass spectrometry (nanoLC–MS/MS). The second method was isobaric chemical labeling using TMT. The 6-plex TMTs were used to label leaf control (TMT^126,127,128^) and ABA-treated samples (TMT^129,130,131^) after phosphopeptide enrichment by IMAC. Protein identification and quantitation were analyzed after LC–MS/MS analysis.

Label-free quantitative proteomic analysis of grapevine leaves in response to ABA was quantified by normalized spectral abundance factors.^28^ Approximately 2533 non-redundant *Vitis vinifera* proteins in the UniProtKB database were identified at low stringency, with 363 and 310 proteins reproducibly identified in samples of leaf control and leaf ABA, respectively, at a FDR of 0.55% (Table 1; Supplementary File 1). Among the total of identified proteins, 20 upregulated and 13 downregulated proteins were significantly differentially expressed in the leaves in response to ABA treatment of the roots (*P*<0.05) (Table 2). A functional analysis for Gene Ontology (GO) categories was analyzed with the Cytoscape (3.2.0, www.cytoscape.org) and the BinGO plugin (3.0.3, www.cytoscape.org) for the statistically significant proteins in response to ABA (Supplementary File 2), using a custom annotation derived from UniProt (uniprot.org), EnsemblPlants (plants.ensembl.org) and Gramene (gramene.org).^33,34^ Photosynthesis, carbohydrate catabolic process and response to abiotic stimulus were significantly over-represented GO categories after correcting for FDR (adjusted *P* value of 0.05) for both significant down- and upregulated proteins in response to ABA. Serine family amino-acid metabolic process was significantly over-represented in downregulated proteins (F6HTU8, F6HTS6, D7SQ37 and D7TAY3), whereas protein folding was significantly over-represented only in upregulated proteins (F6HLR2, D7SJX8 and F6HDM4).

Some of the proteins decreased in abundance by ABA include photosynthetically related proteins such as a photosystem I complex protein (D7TAY3) and a oxygen-evolving enhancer 3 protein, PsbQ (F6H8B4), part of photosystem II. A ribosomal protein (F6HSE3), involved in protein synthesis, was also decreased. Other proteins affected by ABA include proteins involved in amino acid, sugar and cell wall metabolism. A few proteins were increased in protein abundance by ABA including a voltage-dependent anion channel (A5AUG8), an NADP-dependent malic enzyme (P51615) and a putative oxygen-enhancer protein (Q6XGX7).

A total of 1011 proteins were identified by TMT labeling in the leaves of grapevine in response to 10 μm ABA for 2 h at an FDR of 0.62% at the protein level and 0.9% at the peptide level. (Supplementary File 3). A total of 787 proteins were tagged with TMT on the free amino terminus or lysine residues with a 0.61% protein FDR and 0.8% peptide FDR (Supplementary File 4). It should be noted that all proteins reported in this table with quantitation data have been observed in triplicates of both conditions, allowing for statistical evaluation of quantitation differences.

There were 20 proteins (11 phosphoproteins) that were significantly changed in protein abundance (*P*⩽0.05) in the leaves in response to the roots being treated with ABA (Table 3). All significant differentially expressed proteins in response to ABA decreased in protein abundance. Biological process analysis (GO) of significant proteins changing in protein abundance in response to ABA revealed that several developmental processes were affected (*P*⩽0.05; Supplementary File 5). These proteins include the acetyltransferase component of the pyruvate dehydrogenase complex (D7TZW9), a Nck-associated protein 1 (D7T9L3) involved in actin remodeling, a component (F6HTW0) of the Cul4-RING E3 ubiquitin ligase complex, a DNA/RNA helicase (F6GT26), a protein involved in stability of Photosystem II (F6HVA4), a eukaryotic translation initiation factor (F6I2I6) and a ribosomal protein (A5AI30).

### ABA affects phosphoproteins in grapevine

We utilized a new approach for the identification and quantification of phosphoproteins by enrichment of phosphopeptides prior to TMT labeling. This approach gives valuable data on phosphoprotein identification, phosphorylation sites and protein abundance changes. There were 219 phosphoproteins identified by TMT–LC–MS/MS (Supplementary File 6) and 116 of them were tagged with TMT. The phosphoproteins in this table are sorted by the number of discrete peptides identified; 145 proteins were identified from multiple peptides and 74 proteins were identified from single peptides. However, these proteins are not subject to the usual concerns associated with single peptide-based protein identifications because they were actually identified and quantified in triplicate experiments to allow for statistical evaluation.

**Figure 2:**
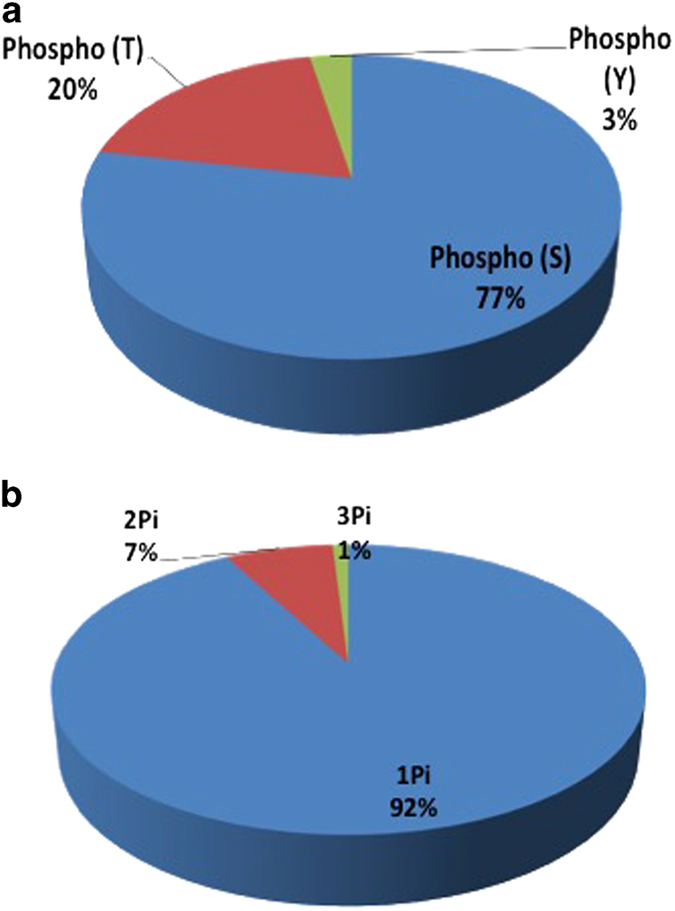
Proportional representation of phosphorylation sites on serine, threonine and tyrosine with Ascores⩾13 (*P*<0.05) found in Cabernet Sauvignon leaf proteins whose roots had been treated with and without 10 μm ABA (**a**) and number of phosphosites, showing that most phosphopeptides were had one phosphosite (92%), followed by two phosphosites (7%) and three phosphosites (1%) (**b**). ABA, abscisic acid.

The phosphoproteins tagged with TMT can be used for further quantification. The localized sites were identified and were reported with Ascore, which calculates the probability of correct phosphorylation site localization based on the presence and intensity of site-determining ions in MS/MS spectra.^35^ From the identified phosphoproteins, 192 non-redundant phosphorylation sites were found with Ascores⩾13 (*P*<0.05) (Supplementary File 7). Identified phosphorylation sites were classified into 77% pSer, 20% pThr and 3% pTyr (Figure 2a). One percent of the proteins had three phosphorylation sites, 7% of the proteins had two phosphorylation sites and 92% of the proteins had one phosphorylation site, (Figure 2b). More than 50% of significant proteins in response to ABA (Table 3) were phosphoproteins. The novel phosphorylation sites with Ascores⩾13 (*P*<0.05) were reported in Supplementary File 7. New phosphorylation sites for many proteins were identified; for example, pectinesterase (F6H777), a potassium efflux antiporter1 (F6I6I6) and an auxin efflux carrier (F6HFI3).

**Figure 3:**
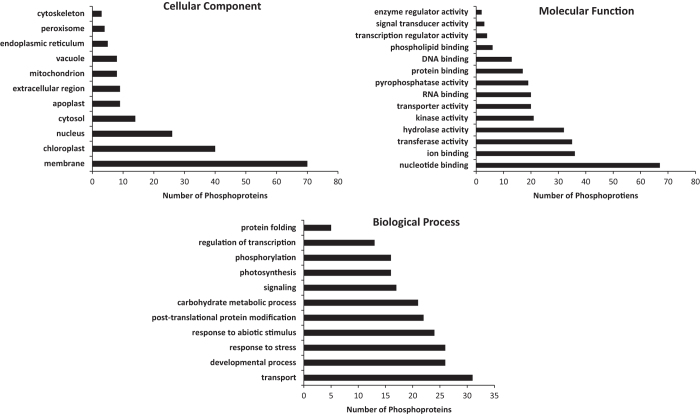
Functional categories of 219 phosphoproteins identified by TMT–LC–MS/MS were analyzed with the Cytoscape (3.2.0) and BinGo plugin (3.0.3). LC–MS, liquid chromatography tandem mass spectrometry; TMT, tandem mass tags.

GO analysis for the phosphoproteins found in this study indicated that nucleotide binding is the most over-represented category of molecular function while membrane and transport is the most over-represented categories of the cellular component and biological process, respectively (Figure 3, Supplementary File 8). The plasma membrane was the highest membrane type for phosphoproteins in this study.

**Figure 4:**
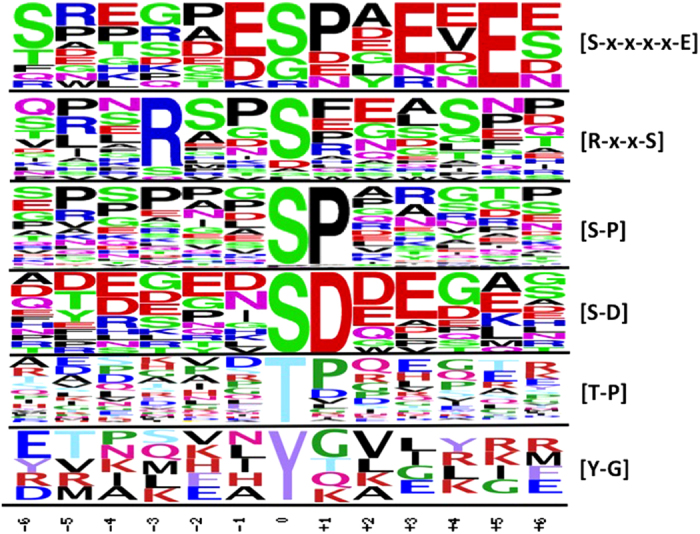
Sequence logos of all phosphorylation sites with Ascores⩾13 (*P*⩽0.05). The frequency of each residue present in each data set is proportional to its height. The phosphorylation site is 0 on the *x* axis flanking by 12 amino-acid residues (at position −6 to +6). Extracted motifs were shown on the right.

The putative motifs surrounding the phosphorylation sites of all phosphopeptides found in this experiment were analyzed with localization probability ⩾95% and Ascores⩾13 (*P*⩽0.05). Finding of motifs involving phosphorylation events provides valuable information about the specific binding of kinases to substrates. The amino acids around the phosphorylation sites from −6 to +6 were aligned to find the common motifs using WebLogo. The motifs found were [S–P], [S–D], [R–x–x–S] and [S–x–x–x–x–E] for phosphoserine, [T–P] for phosphothreonine and [Y–G] for phosphotyrosine (Figure 4; Supplementary File 9).

We also observed a similar response of specific phosphoproteins decreased in protein abundance after treatment with ABA to that which has been previously reported in phosphoproteins in *Arabidopsis;* for example, these included embryonic factor1 (AT2G38280; D7SY29), IQ-domain 32 (AT1G19870; F6H068), seed imbibition 1-like (AT5G40390; D7TWK5) and Tudor/PWWP/MBT protein (AT3G09670; F6HNK4).^11,36^

**Figure 5:**
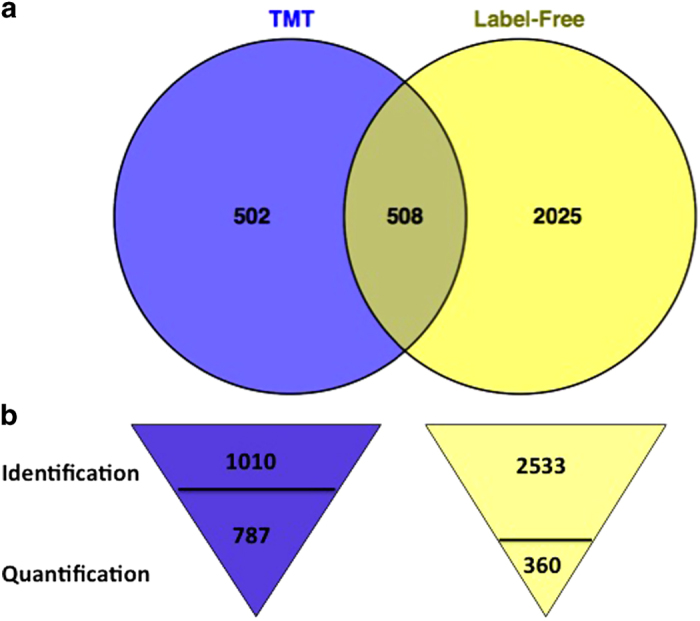
The overlap of proteins found in TMT and label-free method (**a**), and number of protein identification and quantification from TMT and label-free method (**b**).

Furthermore, the same leaf samples were used for all of the transcriptomic, proteomic and phosphoproteomic analysis. Transcriptomic data of ABA-treated leaves was obtained from microarrays,^19^ while proteomic profiles were obtained from TMT and label-free approaches. There were 508 identified proteins found in TMT tagged that were also found in the label-free method (Figure 5a). TMT quantified 787 proteins from 1011 identified proteins, while label-free quantified 360 proteins from 2533 identified proteins (Figure 5b). All three different approaches reported here can identify seven proteins that were found in common (Table 4). Of these seven proteins, just two showed significant effects by ABA, however, their abundance changes were not the same within all three measurement methods. One chlorophyll a/b binding protein (A5BAI4) was increased in protein abundance by ABA based upon the TMT method, but with little or no effect for the label-free and microarray data in the leaves. Interestingly, this protein is phosphorylated. A heat-shock 70 protein, was increased in abundance by ABA in the label-free method, decreased in the TMT method, with little affect on the transcript levels.

## Discussion

### ABA-affected proteins involving plant growth

ABA has multiple physiological effects on plant growth and development. Many of these changes assist the plant to adapt to water deficits, including stomatal closure, photosynthetic protection, antioxidant activities, decreased shoot growth and osmotic adjustment. An increase in endogenous ABA levels is reflective of the degree of water deficit and is normally correlated to growth inhibition.^37,38^ With decreasing soil water potentials, ABA regulates plant growth by inhibiting shoot growth, but promoting root growth.^39^ This is adaptive for the plant resulting in a larger root to shoot ratio, reducing leaf transpiration and increasing water uptake capacity.

Grapevine shoot elongation rate and photosynthesis were inhibited after 4 days of water deficit. However, prior to these physiological changes, there were large changes in protein abundance that were detected.^18^ There was an increase in photosynthetic and antioxidant proteins and a decrease in growth-related proteins for these early changes in response to water deficit. Interestingly, ribosomal proteins involved in protein synthesis were decreased prior to a growth reduction.

The ABA treatment used in this study is likely to represent a concentration of ABA found in leaves exposed to significant water deficit. Our study found a rapid response of proteins to ABA. A number of significant proteins found in our study were involved in organ development. Growth-related proteins were decreased in protein abundance in response to ABA, including several ribosomal proteins, an E2 subunit of the mitochondrial pyruvate dehydrogenase complex (D7TZW9), and a Nck-associated protein 1 (D7T9L3) involved in actin remodeling. A mutant of the gene encoding the E2 subunit of the mitochondrial pyruvate dehydrogenase complex in *Arabidopsis* exhibits retarded growth phenotypes.^40^ Furthermore, ABA inhibits protein synthesis ^23,41^ and growth.^37^ Leaf growth of maize was inhibited under water deficit, which was related to changes of phosphoproteins involved in cell cycle-related processes.^20^ There were significant changes of phosphoproteins in this study that were involved in growth and organ developmental processes. The changes of abundance of these proteins may result in the plant’s acclimation to a drying environment.

Proteins involved in photosynthesis are an important means to control plant growth and development. The inhibition of proteins involved in photosynthesis are correlated with the reduction of shoot elongation of grapevine under water deficit and salinity.^42^ In addition, studies of leaves and fruits treated or affected by ABA showed a decrease in gene expression involved in photosynthesis and a decrease in chlorophyll content.^43–45^ Our study found many significant photosynthetic proteins decreased in protein abundance in response to ABA. Overall, our results indicate that early changes in protein abundance in response to ABA involve plant growth and photosynthesis.

### Phosphoproteomic analysis reveals novel phosphosites and motifs in the ABA signaling pathway

At the time of writing this manuscript, the Plant Protein Phosphorylation Database (P3DB, http://p3db.org), listed 607 phosphoproteins with 862 phosphosites identified in berries of grapevines (*V. vinifera* ‘Italia cv’) using iTRAQ labeling with TiO_2_-phosphopeptide enrichment.^46^ In comparison, we found 219 phosphoproteins with 192 phosphosites in leaves of *V. vinifera* cv. Cabernet Sauvignon using an IMAC-phosphopeptide enrichment and TMT labeling method. In our study, many novel proteins were modified by phosphorylation. The phosphorylation sites found in the previous works^46,47^ were confirmed in this study, such as the phosphosites for ABCG11 (D7T7C0), RuBisCO (F6GWA8) and serine/threonine-protein kinase (F6HPW0). Our study identified five novel pTyr sites with Ascores⩾13 (*P*<0.05) in grapevine proteins. The phosphorylation of tyrosine (Tyr) in plants is less abundant due to the lack of receptor Tyr kinases.^48^ However, *in silico* analysis of the *Arabidopsis* genome indicated that ~4% of Arabidopsis kinases are tyrosine-specific kinases, which was close to the amount of pY found in our study. The proportions of phosphorylation sites on serine, threonine and tyrosine found in this study was consistent with the study on other plant species, such as 89.5% pS, 8.9% pT and 1.6% pY found in rice, and 87.7% pS, 9.9% pT and 2.4% pY found in Arabidopsis.^46,49,50^

We observed a decrease in protein abundance involved in serine family amino-acid metabolic process in leaves in response to ABA. Serines are commonly phosphorylated by kinases during cell signaling. In plants, the phosphorylated pathway of serine biosynthesis has had an important role in supplying serine to non-photosynthetic tissues under environmental stresses.^51,52^

The phosphorylation motifs are important to determine the binding of the kinase to its substrate.^53^ The identification of phosphorylation motifs and phosphorylation site localizations are important in understanding many signal transduction pathways. The motifs found in this study have been identified as the possible substrates of SnRK2s.^10,36^ The SnRK2s have been confirmed to phosphorylate [R–x–x–S] motifs *in vitro.* ^14,54^ We found the phosphorylated [R–x–x–S] motif of the ABC transporter G family member 40 (ABCG40; F6HX69), which decreased in protein abundance in response to ABA. An ABC transporter was found to be able to transport ABA from the cytoplasm to the vacuole in order to control the level of ABA in the cytosol.^55^ ABCG40 is responsible for ABA transport into guard cells in *Arabidopsis.* ^56^ The molecular mechanism of how ABA is transported has not yet been fully elucidated. It is possible that phosphorylation might be involved in this ABA transport mechanism.

Normally, membranes are the first sites of signaling to occur in response to stresses. In this context, many phosphoproteins found in this study are membrane proteins. It has been found that transport systems tend to be phosphorylated,^57,58^ which corresponds to our finding that transport is a major biological process of phosphoproteins. Altogether, these results indicate that ABA may be involved in the regulation of membrane transport systems in grapevine leaves via a protein phosphorylation process.^58^

### Analysis of omics in response to ABA

Label-free and labeling approaches have been found to be equally capable of reliably and accurately quantifying protein abundance levels.^59,60^ Our results confirmed the previous finding^61^ that compared label-free and an isobaric chemical labeling method. Combining label-free with the labeling approach provides a more complete picture for a proteomic study.

On the basis of our results of transcriptomic, proteomic and phosphoproteomic analyses, genes that significantly increased in transcript abundance in response to ABA in the roots, also had their protein abundance changed in the leaves. There might be a signal from the roots causing a change in protein abundance in the leaves. ABA has been proposed as a root-to-shoot signal during drought stress.^62,63^ Our work presented here demonstrated the changing of proteins in the leaves resulting from ABA-treated roots. The protein amino-acid phosphorylation process was a significantly over-represented GO category of DEGs in response to ABA in roots, whereas the organ development process was a significantly over-represented GO category for significant proteins in leaves. More than 50% of significant proteins in response to ABA were phosphoproteins. This finding indicated that there is a communication between roots and leaves in response to ABA, which may involve phosphorylation.

## Conclusions

Utilization of proteomics and phosphoproteomics has provided the data that has lead to deeper understanding of ABA responses in both proteins and phosphorylation of those proteins. With the short-term treatment of ABA, we discovered rapid and significant changes in protein abundance in the leaves of roots treated with exogenous ABA. This finding indicated that there is rapid communication between roots and leaves when responding to ABA. ABA decreased the abundance of growth-related and photosynthetic proteins, probably in an effort to reduce leaf area and water loss. Many potential target proteins and phosphoproteins for ABA signaling were identified. Phosphoproteins found in this study were membrane proteins involved in transport and nucleotide binding. These transport proteins may be involved in plant growth and adaptation to water deficits.

## Figures and Tables

**Table 1:** Peptide/protein identification data of Cabernet Sauvignon leaves: control and ABA

*Condition*	*Low stringency redundant peptide count*			*Low stringency protein identifications*			*High stringency protein identifications*	*Protein FDR (%)*
	*R1*	*R2*	*R3*	*R1*	*R2*	*R3*		
Control	7238	6027	6099	888	840	821	363	0.55
ABA	5269	5650	5715	767	1124	841	310	ND

Abbreviations: FDR, false discovery rate; ND, not detected.

R1, R2 and R3 denote replicate 1, replicate 2 and replicate 3. High-stringency protein indentifications were common to all three replicates.

**Table 2:** Annotation of proteins differentially expressed 2 h after 10 μm ABA treatment measured by a label-free method (method 1)

*Current V1 ID*	*UniProt ID*	*Protein annotation*	*Log*_*2*_ *ratio: ABA/Control*	P *value*
VIT_14s0030g01560	F6HTU8	Cysteine_synthase	−3.59	1.97E−04
VIT_10s0042g00200	F6HIN7	Thioredoxin X	−2.43	4.86E−03
VIT_14s0006g03060	F6HSE3	Ribosomal protein S3, Chloroplast 30S	−2.42	6.31E−03
VITISV_026175	A5B8D1	60S Ribosomal protein L12 family	−1.95	2.42E−04
VIT_05s0094g01380	A5AQ16	Unknown protein	−1.91	8.31E−03
VIT_16s0098g01200	E0CVA1	NagB/RpiA/CoA transferase-like superfamily protein	−1.63	1.49E−02
VIT_11s0052g01710	D7SQ37	Xylose isomerase	−1.31	2.74E−02
VIT_09s0002g06460	D7U0Z4	Alba DNA/RNA-binding protein	−1.26	4.01E−02
VIT_01s0010g03620	D7TAY3	Photosystem I light harvesting complex gene 2	−1.24	2.35E−03
VITISV_041925	A5AEX6	alpha/beta-hydrosolase superfamily protein	−1.07	2.07E−02
VIT_05s0020g02480	D7T6P4	Glutamine_synthetase	−0.84	2.19E−03
VIT_00s0904g00010	F6H8B4	oxygen-evolving enhancer protein 3-2	−0.78	8.20E−03
VITISV_016176	A5BRI2	Protein kinase superfamily protein	−0.74	3.60E−02
VIT_13s0064g01430	D7T2W3	RNA-binding (RRM/RBD/RNP motifs) family protein	−0.69	3.20E−03
VIT_18s0001g00820	F6H0D6	Thioredoxin-like protein CDSP32	−0.43	1.95E−02
VIT_19s0014g03850	A5BX41	Cytochrome_b6f_complex_ironsulfur_subunit	−0.37	1.22E−02
VIT_14s0030g01900	F6HTS6	Ribose-5-phosphate isomerase	−0.35	2.36E−02
VIT_10s0003g02890	A5BAI4	Chlorophyll a/b binding protein 40	0.19	3.68E−02
VIT_12s0028g00320	A5BPB2	Light harvesting chlorophyll-protein complex II subunit B1	0.30	3.16E−02
VIT_19s0014g00160	A5C4U9	Chlorophyll a/b binding protein 1	0.30	2.47E−02
VIT_06s0009g02410	F6HAD6	Elongation factor 1-beta 1	0.68	4.01E−03
VITISV_017201	A5AUG8	Voltage-dependent anion channel 4	0.88	1.62E−02
VITISV_016936	A5BYT5	FRAGILE HISITIDINE TRIAD	1.51	2.89E−02
VIT_05s0020g02880	F6HDM4	Glucose1phosphate adenylyltransferase	2.04	3.71E−02
VIT_06s0004g06610	D7SJX8	Peptidyl-prolyl *cis–trans* isomerase	2.08	3.76E−02
VIT_11s0016g03210	P51615	NADP-dependent malic enzyme	2.59	5.00E−02
VITISV_008240	A5BGC9	6-phosphogluconate dehydrogenase, decarboxylating	2.73	3.95E−03
VIT_18s0072g01000	F6GY10	2-oxoacid dehydrogenase acyltransferase family protein	2.79	1.95E−02
VIT_14s0030g02180	F6HTR2	GDP-mannose 3,5-epimerase 1	2.79	1.95E−02
VIT_10s0003g03260	F6HLR2	Prefoldin 6	2.96	1.26E−03
VITISV_033715	A5AZX9	Triosephosphate isomerase	3.16	1.27E−04
VITISV_033255	A5AFH5	Cysteine synthase	3.96	1.16E−03
VIT_13s0019g00260	Q6XGX7	Putative oxygen-evolving enhancer protein	4.00	2.30E−05

**Table 3:** Annotation of proteins differentially expressed 2 h after 10 μm ABA treatment by TMT

*VIT ID*	*Uniprot ID*	*Annotation*	*Log*_*2*_ *ratio: ABA/control*	P *value*	*Peptide sequence*	*Variable modification*
VIT_14s0066g01120	F6HUY8	Tudor/PWWP/MBTsuperfamilyprotein	−1.59	1.12E−04	GNEAESHVVNSNLAsPR	S526 Phospho
VIT_14s0030g01350	F6HTW0	Transducin/WD40 repeat-like superfamily protein	−1.66	6.69E−04	VGSAGNTSNsTRPR	S18 Phospho
					VGSAGNTSNsTRPR	S20 Phospho
VIT_17s0000g06950	F6GT26	RNA helicase family protein	−1.24	6.51E−03	TSQDEDDDsELEEESLRDR	S173 Phospho
VITISV_031115	A5AI30	Ribosomal protein S21 family protein	−1.83	1.17E−02	NKKDDDEEDNWEVPEGELPF	
VITISV_013443	D7U6G6	Anthocyanidin reductase	−1.54	1.23E−02	YGIEEIYDESVEYFK	
VITISV_040194	A5BVL2	Hypothetical protein VITISV_040194	−0.88	1.28E−02	HRPSSPQPPPPPPPQR	
VIT_12s0028g03150	F6H5G6	Nuclear cap-binding protein subunit 2	−2.43	1.38E−02	FRESGDsDDEEEDDR	S112 Phospho
VIT_04s0008g05880	F6H3J1	PHD finger family protein	−0.90	1.43E−02	SDRRPIYNLDEsDDDADLVHGK	S23 Phospho
VIT_00s0361g00080	F6I216	Eukaryotic translation initiation factor-related	−0.73	1.45E−02	ERNPQSYNDGVQVsPTNGK	S330 Phospho
					ERNPQSYNDGVQVSPtNGK	T332 Phospho
VIT_13s0019g03620	F6HNK4	Tudor/PWWP/MBT superfamily protein	−0.86	1.46E−02	DHNDACVsPDERTQVAER	S509 Phospho
VIT_07s0005g02360	F6HZE7	CONTAIN Hepatocellular carcinoma-associated antigen 59 domain	−0.99	1.58E−02	SIEDDQAKDNNNSEDEEERR	
VIT_01s0011g01630	D7T9L3	Transcription activators	−1.32	1.64E−02	QHFANQDASLsPTAGR	S16 Phospho
VIT_09s0002g01800	D7TZW9	Dihydrolipoamide acetyltransferase	−1.61	2.03E−02	VGEVIAITVEEEEDIAKFK	
VIT_01s0011g00820	D7T9T5	Remorin family protein	−1.19	2.28E−02	TTPPPPPPPPPPPPsVQKTPTVK	S163 Phospho
					TTPPPPPPPPPPPPSVQKTPtVK	T169 Phospho
VIT_05s0094g01520	D7T2N7	Late embryogenesis abundant protein, group 2	−1.50	2.45E−02	DKGVGEDDDDDED	
VIT_13s0084g00160	F6HVA4	Proline-rich family protein	−1.31	2.69E−02	ASSDDSDCNDEECAPDKEVGK	
VIT_06s0009g02120	D7T1D7	dr1-associated corepressor	−1.31	2.73E−02	VVDDEGNDSDEESkR	S116 Phospho
VIT_10s0003g04630	D7TKH5	SIT4 phosphatase-associated family protein	−0.78	3.37E−02	TRDSDDDDYQDRDYDVAALANNLSQAFR	
VIT_18s0001g01180	F6H0F0	Target of MYB protein 1	−0.36	4.30E−02	GAPAVAVGTTESSAPVLVNVTHEDDEsEDDFAQLAHR	S293 Phospho
VIT_15s0046g00490	F6I6E1	*O*-acyltransferase (WSD1-like) family protein	−0.38	4.96E−02	SAGREVEGDGEKPEDIEEEEEPLSPAAR	

**Table 4:** Common proteins found from transcriptomic, proteomic and phosphoproteomic analysis. The microarray data is from a previous publication^19^

*Current V1 ID*	*UniProt ID*	*Annotation*	*Microarray*		*Label-free*	*TMT*	*Phosphorylated*
			*Log*_*2*_ *LA/LC*	*Log*_*2*_ *RA/RC*	*Log*_*2*_ *LA/LC*	*Log*_*2*_ *LA/LC*	
VIT_10s0003g02890	A5BAI4	Chlorophyll a/b binding protein 3	−0.045	2.133	0.188	0.934	Yes
VIT_06s0061g00270	F6GWA8	Chaperonin 60 subunit alpha 1, chloroplastic	0.062	1.431	0.321	−0.363	Yes
VIT_18s0001g02740	E0CR63	Photosystem II 22 kDa	−0.024	2.042	0.028	−0.011	Yes
VIT_08s0007g00130	F6HLD8	Heat-shock protein 70	−0.111	1.433	2.214	−0.725	
VIT_14s0060g00820	A5AIE0	Chloroplast stem-loop binding protein of 41 kDa b, chloroplastic	0.036	1.284	0.291	−0.561	
VIT_06s0004g00240	D7SLM9	Chaperonin 60 subunit beta 3, chloroplastic	−0.044	1.050	0.015	−0.216	
VIT_15s0024g00040	F6I519	Photosystem I light harvesting complex gene 3	0.090	1.705	−0.385	0.234	
